# Colorectal cancer survival disparities in the five regions of Georgia

**DOI:** 10.1371/journal.pone.0301027

**Published:** 2024-03-28

**Authors:** Meng-Han Tsai, Daramola N. Cabral, Caitlyn Grunert, Justin X. Moore

**Affiliations:** 1 Cancer Prevention, Control, & Population Health Program, Georgia Cancer Center, Augusta University, Augusta, Georgia, United States of America; 2 Georgia Prevention Institute, Augusta University, Augusta, Georgia, United States of America; 3 Department of Health, Human Services, and Public Policy, College of Health Sciences and Human Services, California State University, Monterey Bay, Seaside, California, United States of America; 4 African Caribbean Cancer Consortium, Philadelphia, Pennsylvania, United States of America; 5 Department of Health Management and Policy, University of Kentucky, Lexington, Kentucky, United States of America; 6 Center for Health Equity Transformation, Department of Behavioral Science, Department of Internal Medicine, Markey Cancer Center, University of Kentucky College of Medicine, Lexington, Kentucky, United States of America; University of Tennessee at Chattanooga, UNITED STATES

## Abstract

**Background/objective:**

The objective of this study was to examine 5-year colorectal cancer survival rates. We also determined whether demographics, tumor characteristics, and treatment modality were associated with 5-year CRC survival in the Clayton, West Central, East Central, Southeast, and Northeast Georgia regions because the significant higher CRC mortality rates in these regions in comparison to the overall rates in the State of Georgia.

**Methods:**

We conducted a retrospective cohort analysis using data from the 1975–2016 Surveillance, Epidemiology, and End Results program aggregated CRC patients to these five regions. Five-year CRC survival was calculated and stratified by the five regions of Georgia, using the Kaplan-Meier method with log-rank test. Cox proportional hazard regression was used to examine the mentioned association in these five regions.

**Results:**

Among 11,023 CRC patients, 5-year CRC survival was lowest in Clayton (65.9%) compared to the West Central (69.0%), East Central (68.2%), Southeast (70.5%), and Northeast regions (69.5%) (p-value = 0.02). In multivariable analysis, greater risk of CRC death was found in the Clayton region compared to the West Central (HR, 1.12; 95%, 1.00–1.25) region when adjusting for demographics, tumor characteristics, and treatment modality. Among Clayton Georgians, age of 75+ years (HR, 2.13; 95%, 1.56–2.89), grade 3 & 4 tumors (HR, 2.22; 95%, 1.64–3.00), and distant stage (HR, 20.95; 95%, 15.99–27.45) were negatively associated with CRC survival.

**Conclusion:**

We observed place-based differences in CRC survival with significantly lower survival rates in the Clayton region. Factors associated with higher risk of CRC death include older age at diagnosis, high-grade tumors, and distant stage CRC among Clayton Georgians. Our study provides important evidence to all relevant stakeholders in furthering the development of culturally tailored CRC screening interventions aimed at CRC early detection and improved outcomes.

## Introduction

In Georgia, colorectal cancer (CRC) is the second leading cause of cancer morbidity and mortality despite an annual reduction in mortality rates of 2.3% per year during 2002–2013 [1]. However, CRC mortality rates vary across different regions of Georgia. Prior studies suggests that there are significantly higher CRC mortality rates observed in Clayton, East Central, West Central, Northeast, and Southeast regions of Georgia compared with the overall mortality rate in Georgia from years 2008 through 2013 [1]. Barriers to detection and prevention, such as lack of patient awareness, patient and provider communication, and high out of pocket healthcare costs have been reported as important factors linked to delayed screening or treatment of CRC [2, 3], which ultimately affect CRC survival outcomes.

Further, lower socioeconomic status and lack of health insurance are also important social determinants of health that drive poorer survival among CRC patients [4]. From 2019 to 2021, a significantly higher poverty rate was observed in Georgia compared to the United States (US) national average (13.1% vs. 11.6%) [5]. Several Georgian counties within the mentioned regions, such as Clayton, Richmond (East Central region), and Muscogee (West Central region) counties, are currently considered as lower socioeconomic areas [6]. Several of these counties are characterized by predominantly Black population, with 19% to 22% of the population living in poverty [6]. More importantly, around 19% of Georgia residents have higher uninsured rates compared to the national rate of 12.6% [7], which is strongly associated with poor outcomes for CRC patients [8]. Therefore, such findings highlight the need for evaluating CRC survival and risk profiles among Georgians living in socioeconomically disadvantaged regions while focusing on reducing CRC mortality through timely screening and treatment.

Factors associated with survival outcome include patient demographics, tumor characteristics, and treatment modality. A prior study reported that adults aged 65 years or older had a 28% lower CRC 5-year survival rate compared to adults younger than 65 years old [9]. Gender differences in 5-year CRC survival rates have also been observed, with survival rates of 50% in women compared to 44% in men [10]. Other demographic factors associated with poorer CRC survival include non-Hispanic Black race and being unmarried [11]. Additionally, late-stage CRC is associated with a significant reduction in survival of CRC [1, 9, 12, 13]. Effective chemotherapy regimens has increased the median survival to more than 3 years for CRC patients despite late-stage diagnosis [12].

To date, studies that have examined 5-year survival rates and determinants of CRC survival primarily focus on the US population [14] or larger geographic areas [15]. Limited research has examined CRC rates and predictors in Georgia [16], particularly comparisons between small geographic areas by using multi-year registries. Only a recent ecological study examined geographic variation in cancer mortality within the state of Georgia without considering cancer-specific survival at the individual-level [17]. A detailed analysis of CRC survival profiles considering demographic data, tumor characteristics, and treatment modality within the small geographic areas is essential to guide future studies on CRC etiopathogenesis. Studies to examine multifaceted factors (e.g., lifestyle and environmental factors) are needed to inform local CRC interventions and screening initiatives for early detection. To address these research gaps, our study aimed to 1) examine the 5-year survival rates of CRC and 2) determine the factors associated with CRC survival in Clayton, East Central, West Central, Northeast, and Southeast regions of Georgia by using population-based cancer registry.

## Methods

### Study design, data sources, and study participants

We conducted retrospective cohort analysis using data from the 1975–2016 Surveillance, Epidemiology, and End Results (SEER) Program (November 2018 submission), which are sources for comprehensive population-based information in the US that includes patient demographics, primary tumor site, tumor morphology and stage at diagnosis, first course of treatment, and follow-up for vital status. The study eligible population included patients diagnosed with CRC defined by the SEER Site Recode ICD-O-3/WHO 2008 definition of colon cancer (C180–C189), rectosigmoid junction cancer (C199), and rectal cancer (C209) [18]. In addition, we used the county Federal Information Processing System (FIPS) code for the State of Georgia “13” and county for coding these five regions of Georgia [18, 19]. Further, we identified CRC patients who lived in those counties within the five regions of interest and aggregated individual-level data to these five regions using the definition of Georgia public health districts [1]. These five regions include one county in the Clayton,13 counties in the East Central, 16 counties in the West Central, 22 counties in the Southeast, and 10 counties in the Northeast regions [1]. Data extracted for this study were publicly available and de-identified, and thus considered exempt from institutional review board (IRB) review.

### Study eligible participants

**Fig 1:**
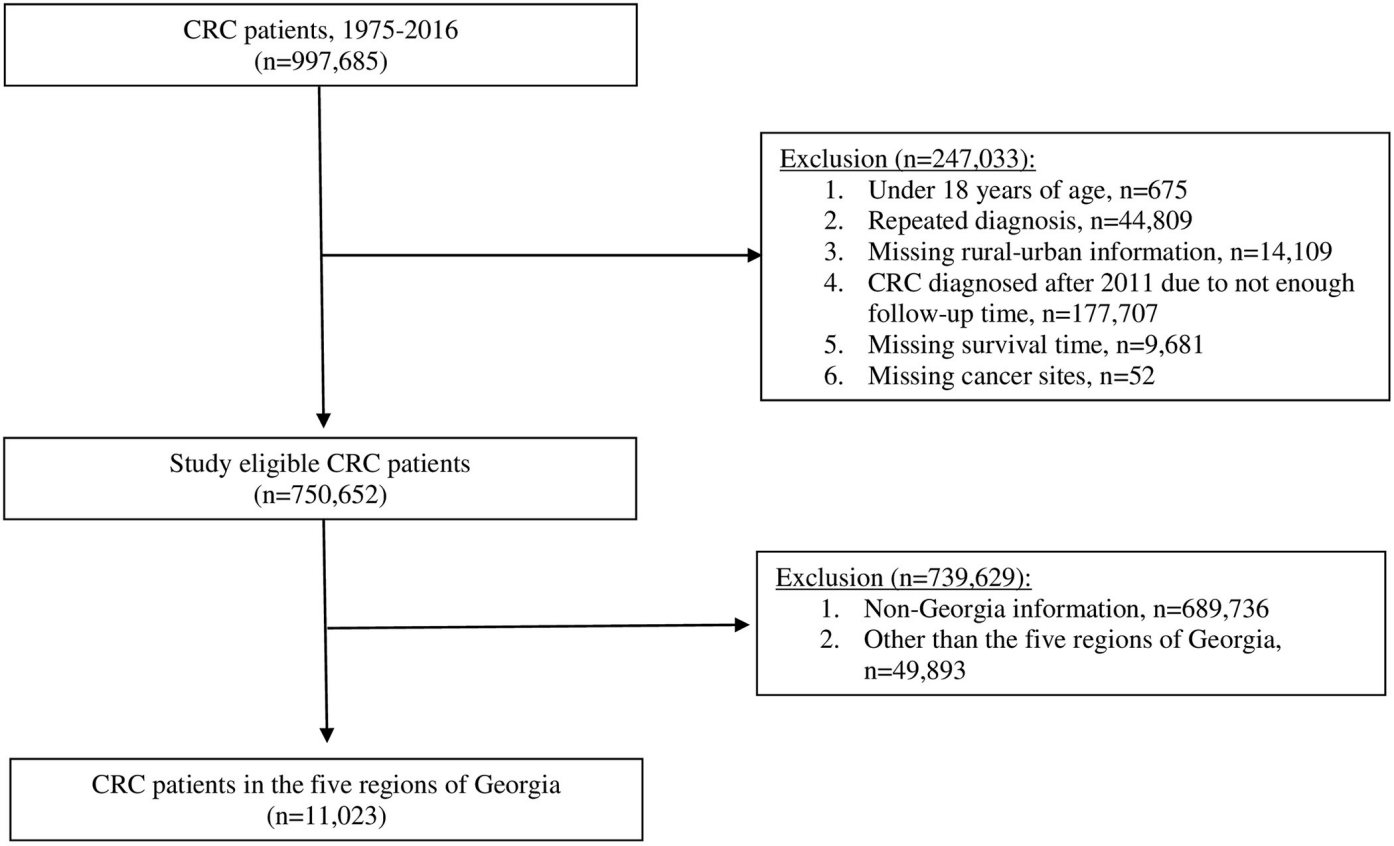
Flowchart of eligible participants. Abbreviation: CRC, colorectal cancer.

A total of 997,685 CRC patients were included in SEER for 1975–2016. To obtain an eligible study sample, we excluded 247,033 CRC patients aged under 18 years (n = 675), repeated diagnosis of CRC (n = 44,809), missing rural and urban information (n = 14,109), CRC diagnosed after 2011 due to limited follow-up time (less than five years) (n = 177,707), missing survival time (n = 9,681), and missing cancer sites (n = 52). Further, we excluded 739,629 CRC patients who did not live in Georgia (n = 689,736) and the five regions of interest (n = 49,893) (Fig 1). Our rationale of certain exclusion criteria, such as CRC patients aged < 18 years, repeated diagnoses, and missing rural/urban information, are described. First, we excluded patients aged < 18 years because cancer prognosis is different between children, adolescents, and adults. Second, there were some repeated records from the same patients and diagnosis; thus, we excluded them from analysis. Finally, we excluded missing rural and urban information because this is an important factor and could have potential impact on CRC outcomes due to unequal access to treatment and screening facilities. As a result, 11,023 CRC patients living in Clayton, West Central, East Central, Southeast, and Northeast regions of Georgia were included in the analyses.

### Measures: Outcome, exposure, and covariates

CRC survival was the outcome of interest, and region (Clayton, West Central, East Central, Southeast, and Northeast regions) was our primary risk factor. Other covariates of interest included are socio-demographics, tumor characteristics, treatment modality, and year of diagnosis. Those covariates were adjusted in multivariable models and evaluated for their impact on CRC survival. For demographic characteristics, we included age category at diagnosis (18–44, 45–54, 55–64, 65–74, or ≥ 75), gender (male or female), race (White, Black, or Other), marital status (single, married, others, or unknown), and rurality (urban or rural). In tumor characteristics, we included grade (grade 1, 2, 3 &4, or unknown), stage at diagnosis (localized, regionalized, distant, or unknown), and primary site (right or left). Patients’ first course of treatment modality, chemotherapy (yes or no/unknown) and radiation (yes or no/unknown) were also included. Finally, we also included year of diagnosis (1975–1984, 1985–1994, 1995–2004, or 2005+) as one of covariates.

### Statistical analysis

Descriptive statistics were used to describe the distribution of CRC patients within the five regions of Georgia, including demographics, tumor characteristics, treatment modality, and year of diagnosis. We compared bivariate differences across five Georgia regions in demographics, tumor characteristics, treatment modalities, and year of diagnosis, using chi-square test for categorical variables and analysis of variance (ANOVA) for continuous variables. Patients’ survival time was measured in months from the date of diagnosis up to 60-months of follow up, censored at the end of study observation period (December 31, 2016) or death. Survival analysis at five-year interval was performed using the Kaplan-Meier method. The Log-rank test was used to compare the survival rates within the five regions of Georgia. Further, we performed Cox proportional hazard regression to examine the impact of place-based differences on CRC survival in these five regions. Three sequential models were performed to examine this association. Model 1 was adjusted for demographic characteristics (age at diagnosis, gender, race, marital status, rurality); model 2 was further adjusted for tumor characteristics (grade, stage, primary site) and treatment modality (chemotherapy, radiation); model 3 was further adjusted for year of diagnosis. Finally, the sub-population analyses within each region of Georgia were also preformed to determine the factors (demographic characteristics, tumor characteristics, and treatment modality) associated with CRC survival by using the Cox proportional hazard regression adjusting for year of diagnosis. The level of statistical significance was set at an alpha level of 0.05 and the p-values were based on two-sided probability tests. We used SAS Version 9.4, SAS Institute Inc., Cary, North Carolina, and Stata Version 16 (Stata Corporation LLC, College Station, TX, USA) to perform analyses.

## Results

### Patient demographics, tumor characteristics, and treatment modality

Table 1 describe the demographic characteristics, tumor characteristics, treatment modality, and year of diagnosis stratified by the selected five regions. For demographic characteristics, most patients were aged 55–64 in the Clayton region and were aged 75 years or older in the East Central, West Central, Southeast, and Northeast regions. The average age at diagnosis was 63.1 years (SD, 13.3) in Clayton, which was younger than the other four regions. There was an equal proportion of male and female patients for the Clayton and West Central regions; however, 52.7%-53.7% of the East Central, Southeast, and Northeast Georgians were females. Over half of CRC patients were White (particularly in the Southeast and Northeast regions), married, and living in urban areas (except for the Southeast region) for these five regions. Further, tumor characteristics demonstrated different distribution across these five regions (p-value <0.05). Results show that over 50% of the Georgians were diagnosed with grade 2 for these five regions. Most patients were diagnosed with localized CRC for the West Central, East Central, and Southeast regions. In Clayton and Northeast regions, most patients were diagnosed with regional CRC. Over half of the CRC diagnoses was found in left colon and over 60% of Georgians were treated without using chemotherapy in these selected regions. Finally, majority of CRC diagnoses were observed in year 2005 or later for these five regions.

**Table 1:** Distribution of patients within five regions of Georgia, demographic characteristic, tumor characteristics, and treatment modality (n = 11,023).

	Total (n = 11,023)	Clayton (n = 2,001)	West Central (n = 2,027)	East Central (n = 2,578)	Southeast (n = 2,158)	Northeast (n = 2,259)	P-value
	n (%)	n (%)	n (%)	n (%)	n (%)	n (%)	
**Demographic characteristics**							
**Age at diagnosis, mean (SD)**	65.3	63.1	66.6	65.5	65.2	66.0	<0.001
	(SD, 13.4)	(SD, 13.3)	(SD, 13.2)	(SD, 13.3)	(SD,13.2)	(SD,13.9)	
18–44	689(6.3%)	176(8.8%)	87(4.3%)	150(5.8%)	130(6.0%)	146(6.5%)	
45–54	1,793(16.3%)	350(17.5%)	325(16.0%)	422(16.4%)	357(16.5%)	339(15.0%)	
55–64	2,679(24.3%)	558(27.9%)	454(22.4%)	630(24.4%)	522(24.2%)	515(22.8%)	
65–74	2,816(25.6%)	490(24.5%)	526(26.0%)	661(25.6%)	568(26.3%)	571(25.3%)	
≥75	3,046(27.6%)	427(21.3%)	635(31.3%)	715(27.7%)	581(26.9%)	688(30.5%)	
**Gender**							0.029
Male	5,753(52.2%)	1,014(50.7%)	1,018(50.2%)	1,219(47.3%)	1,000(46.3%)	1,046(46.3%)	
Female	5,270(47.8%)	987(49.3%)	1,009(49.8%)	1,359(52.7%)	1,158(53.7%)	1,213(53.7%)	
**Race**							<0.001
White	7,507(68.1%)	1,336(66.8%)	1,200(59.2%)	1,520(59.0%)	1,673(77.5%)	1,778(78.7%)	
Black	3,369(30.6%)	619(30.9%)	809(39.9%)	1,022(39.6%)	465(21.6%)	454(20.1%)	
Other	147(1.3%)	46(2.3%)	18(0.9%)	36(1.4%)	20(0.9%)	27(1.2%)	
**Marital status**							0.001
Single	1,349(12.2%)	241(12.0%)	278(13.7%)	330(12.8%)	265(12.3%)	235(10.4%)	
Married	5,958(54.1%)	1,121(56.0%)	1,026(50.6%)	1,346(52.2%)	1,194(55.3%)	1,271(56.3%)	
Others ^a^	3,294(29.9%)	559(27.9%)	638(31.5%)	787(30.5%)	644(29.8%)	666(29.5%)	
Unknown	422(3.8%)	80(4.0%)	85(4.2%)	115(4.5%)	55(2.6%)	87(3.9%)	
**Rurality**							<0.001
Urban	7,304(66.3%)	2,001(100%)	1,344(66.3%)	1,840(71.4%)	496(23.0%)	1,623(71.9%)	
Rural	3,719(33.7%)	0	683(33.7%)	738(28.6%)	1,662(77.0%)	636(28.2%)	
**Tumor characteristics**							
**Grade** ^b^							<0.001
Grade 1	1,229(11.2%)	286(14.3%)	218(10.8%)	263(10.2%)	160(7.4%)	302(13.4%)	
Grade 2	6,598(59.9%)	1,087(54.3%)	1,326(65.4%)	1,575(61.1%)	1,287(59.6%)	1,323(58.6%)	
Grade 3 &4	1,439(13.1%)	278(13.9%)	223(11.0%)	311(12.1%)	288(13.4%)	339(15.0%)	
Unknown	1,757(15.9%)	350(17.5%)	260(12.8%)	429(16.6%)	423(19.6%)	295(13.1%)	
**Stage**							<0.001
Localized	4,237(38.4%)	723(36.1%)	804(39.7%)	984(38.2%)	903(41.8%)	823(36.4%)	
Regionalized	4,044(36.7%)	761(38.0%)	738(36.4%)	925(35.9%)	715(33.1%)	905(40.1%)	
Distant	2,171(19.7%)	412(20.6%)	414(20.4%)	499(19.4%)	408(18.9%)	438(19.4%)	
Unknown	571(5.2%)	105(5.3%)	71(3.5%)	170(6.6%)	132(6.1%)	93(4.1%)	
**Primary site** ^c^							0.028
Right	4,295(39.0%)	742(37.1%)	846(41.7%)	1,005(39.0%)	815(37.8%)	887(39.3%)	
Left	6,728(61.0%)	1,259(62.9%)	1,181(58.3%)	1,573(61.0%)	1,343(62.2%)	1,372(60.7%)	
**Treatment modality**							
**Chemotherapy**							<0.001
No/Unknown	7,350(66.7%)	1,455(72.7%)	1,224(60.4%)	1,760(68.3%)	1,431(66.3%)	1,480(65.5%)	
Yes	3,673(33.3%)	546(27.3%)	803(39.6%)	818(31.7%)	727(33.7%)	779(34.5%)	
**Radiation**							0.134
No/Unknown	9,750(88.5%)	1,774(88.7%)	1,765(87.1%)	2,299(89.2%)	1,897(87.9%)	2,015(89.2%)	
Yes	1,273(11.6%)	227(11.3%)	262(12.9%)	279(10.8%)	261(12.1%)	244(10.8%)	
**Year of diagnosis**							
1975–1984	319(2.9%)	319(15.9%)	0	0	0	0	<0.001
1985–1994	546(5.0%)	472(23.6%)	0	42(1.6%)	0	32(1.4%)	
1995–2004	4,460(40.5%)	602(30.1%)	863(42.6%)	1,135(44.0%)	915(42.4%)	945(41.8%)	
2005+	5,698(51.7%)	608(30.4%)	1,164(57.4%)	1,401(54.3%)	1,243(57.6%)	1,282(56.8%)	

Abbreviation: mos., months; SD, standard deviation.

^a^ Others include divorced, separated, and widow.

^b^ Grade 1:Well differentiated; Grade 2: moderately differentiated; Grade 3: poorly differentiated; Grade 4: undifferentiated.

^c^ Right: cecum to transverse; left: splenic flexure to rectum.

### Five-year survival rates

**Fig 2:**
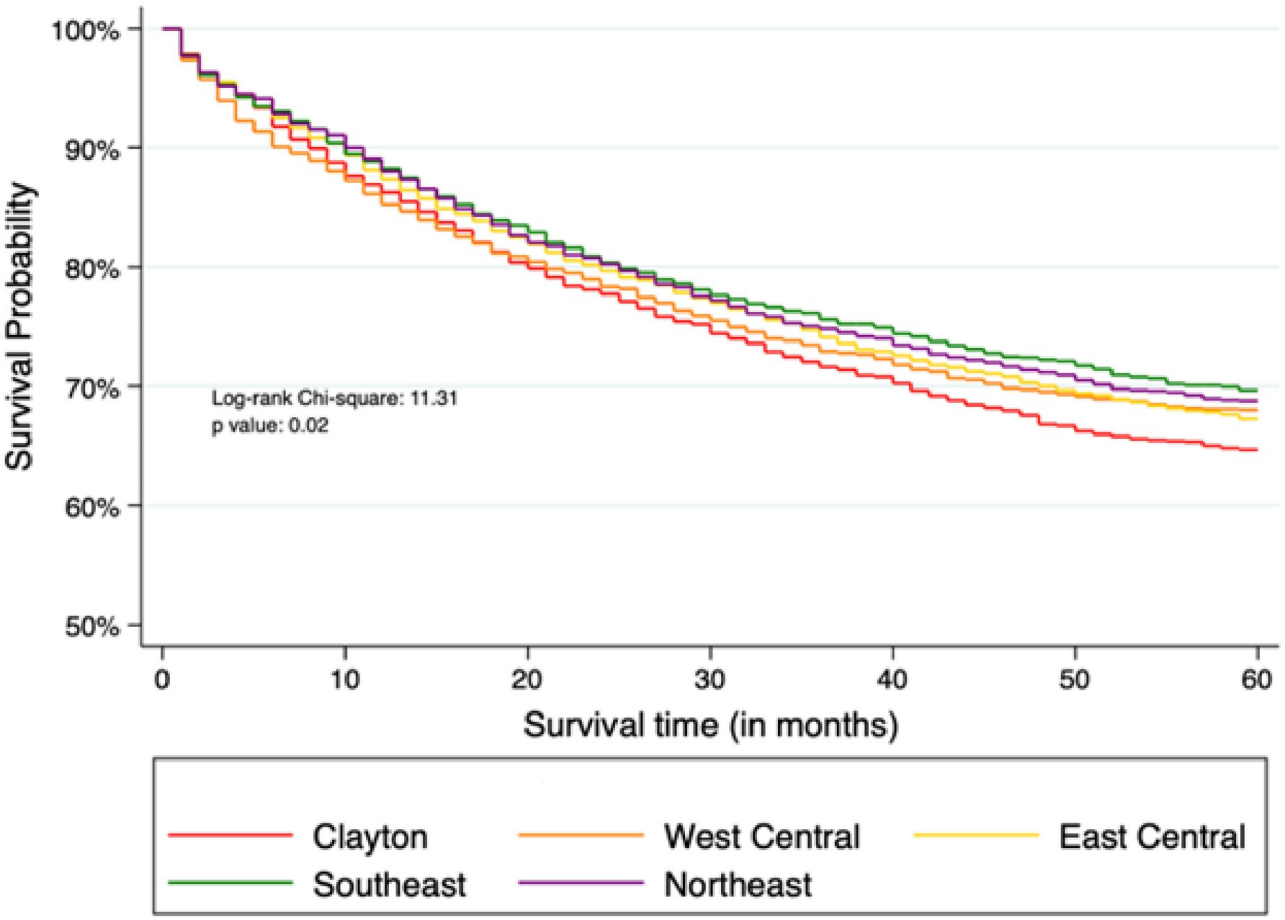
Kaplan Meier CRC survival curves in the Clayton, West Central, East Central, Southeast, and Northeast of Georgia regions. Abbreviation: CRC, colorectal cancer.

The mean survival time since CRC diagnosis was 71.7 months (SD, 65.5 months) and the overall five-year survival rate was 68.6% (95% CI, 67.6%-69.5%) in these five regions of Georgia. When exploring the survival rates within each region, the five-year survival rates were 65.9% (95% CI, 63.7%-67.9%) in the Clayton, 69.0% (95% CI, 66.9%-71%) in the West Central, 68.2% (95% CI, 66.4%-70%) in the East Central, 70.5% (95% CI, 68.5%-72.4%) in the Southeast, and 69.5% (95% CI, 67.5%-71.3%) in the Northeast regions (p-value <0.05) (Fig 2).

### Determinants of colorectal cancer survival

In Table 2, results show that the Clayton Georgians (HR, 1.22; 95% CI, 1.09–1.36) demonstrated greater CRC risk of death compared to the West Central Georgians when adjusting for demographic characteristics. Similarly, Clayton Georgians had 1.1-fold increased risk on CRC death when adjusting for demographics, tumor characteristics, and treatment modality (HR, 1.12; 95% CI, 1.00–1.25). Further, we examined whether demographics, tumor characteristics, and treatment modality associated with risk of CRC death stratified by the five selected regions (Table 3). Age at diagnosis was positively associated with CRC death regardless of the regions of Georgia, particularly for age of 75 years or older. Female CRC patients were 15% -22% of less likely of CRC death in the Clayton, East Central, Southeast, and Northeast regions. When exploring tumor characteristics, tumor grades 3 and 4 were positively associated with CRC death in all five regions, with 1.5–2.5-fold of increased risk of CRC death. Moreover, having distant stage of CRC were associated with 16.5–22.5-fold of increased risk of CRC death in these selected regions. Particularly, East Central (HR, 22.50; 95% CI, 17.55–28.83) Georgians had greater risk of CRC death when diagnosed with distant CRC compared to those diagnosed localized cancer. Finally, receipt of chemotherapy was associated with lower risk of CRC death in the West Central (HR, 0.80; 95% CI, 0.66–0.97), East Central (HR, 0.78; 95% CI, 0.66–0.93), and Southeast (HR, 0.71; 95% CI, 0.59–0.86) regions.

**Table 2:** Determinants of CRC risk of death in the five regions of Georgia (n = 11,023).

	Model 1		Model 2		Model 3	
	HR (95%CI)	P-value	HR (95%CI)	P-value	HR (95%CI)	P-value
**Five Georgia regions**		<0.001		0.008		0.431
West Central	Reference		Reference		Reference	
Clayton	*1*.*22(1*.*09*,*1*.*36)*		*1*.*12(1*.*00*,*1*.*25)*		1.01(0.89,1.15)	
East Central	1.02 (0.92,1.13)		0.99(0.89,1.10)		0.99(0.89,1.10)	
Northeast	0.99(0.90,1.12)		0.93(0.83,1.04)		0.94(0.84,1.05)	
Southeast	0.94(0.83,1.05)		0.91(0.81,1.02)		0.91(0.81,1.03)	

Abbreviation: CRC, colorectal cancer; HR, hazard ratio. Notes: 1) Italicized text indicates statistically significant result; 2) Model 1 was adjusted for demographic characteristics; model 2 was further adjusted for tumor characteristics and treatment modality; model 3 was further for year of diagnosis.

^a^ Others include divorced, separated, and widow.

^b^ Grade 1: Well differentiated; Grade 2: moderately differentiated; Grade 3: poorly differentiated; Grade 4: undifferentiated.

^c^ Right: cecum to transverse; left: splenic flexure to rectum.

**Table 3:** Determinants of CRC risk of death stratified by the five regions of Georgia (n = 11,023).

	Clayton	West Central	East Central	Southeast	Northeast
	HR (95%CI)	HR (95%CI)	HR (95%CI)	HR (95%CI)	HR (95%CI)
**Demographic characteristics**					
**Age at diagnosis**					
18–44	Reference	Reference	Reference	Reference	Reference
45–54	1.01(0.75,1.37)	0.95(0.60,1.51)	1.39(0.99,1.94)	0.81(0.55,1.20)	1.04(0.74,1.45)
55–64	1.05(0.79,1.41)	1.22(0.78,1.91)	1.31(0.95,1.81)	0.90(0.62,1.30)	1.09(0.79,1.51)
65–74	*1*.*56(1*.*16*,*2*.*09)*	1.40(0.90,2.17)	*1*.*44(1*.*04*,*2*.*01)*	1.17(0.80,1.70)	1.32(0.95,1.83)
≥75	*2*.*13(1*.*56*,*2*.*89)*	*2*.*16(1*.*39*,*3*.*36)*	*2*.*14(1*.*53*,*2*.*98)*	*2*.*04(1*.*41*,*2*.*94)*	*2*.*29(1*.*65*,*3*.*18)*
**Gender**					
Male	Reference	Reference	Reference	Reference	Reference
Female	*0*.*82(0*.*70*,*0*.*96)*	0.85(0.72,1.00)	*0*.*85(0*.*74*,*0*.*98)*	*0*.*81(0*.*69*,*0*.*96)*	*0*.*78(0*.*66*,*0*.*92)*
**Race**					
White	Reference	Reference	Reference	Reference	Reference
Black	*1*.*24(1*.*01*,*1*.*52)*	*1*.*24(1*.*05*,*1*.*46)*	1.01(0.87,1.17)	1.04(0.86,1.26)	*1*.*28(1*.*07*,*1*.*54)*
Other	0.84(0.47,1.51)	0.68(0.17,2.77)	0.68(0.34,1.38)	0.71(0.29,1.74)	1.17(0.55,2.48)
**Marital status**					
Single	Reference	Reference	Reference	Reference	Reference
Married	0.87(0.68,1.11)	*0*.*70(0*.*56*,*0*.*89)*	*0*.*76(0*.*61*,*0*.*94)*	*0*.*69(0*.*54*,*0*.*88)*	*0*.*70(0*.*54*,*0*.*90)*
Others ^a^	1.08(0.83,1.41)	0.93(0.73,1.18)	0.99(0.79,1.26)	0.83(0.64,1.07)	1.01(0.77,1.32)
Unknown	0.93(0.68,1.11)	0.67(0.39,1.17)	0.88(0.60,1.28)	0.77(0.43,1.36)	1.04(0.68,1.58)
**Rurality**					
Urban	NA^d^	Reference	Reference	Reference	Reference
Rural	NA^d^	1.06(0.89,1.25)	*1*.*23(1*.*05*,*1*.*43)*	0.98(0.81,1.18)	1.01(0.85,1.19)
**Tumor characteristics**					
**Grade** ^b^					
Grade 1	Reference	Reference	Reference	Reference	Reference
Grade 2	*1*.*34(1*.*03*,*1*.*75)*	0.92(0.68,1.25)	*1*.*38(1*.*00*,*1*.*89)*	1.22(0.83,1.81)	*1*.*43(1*.*06*,*1*.*94)*
Grade 3&4	*2*.*22(1*.*64*,*3*.*00)*	*1*.*45(1*.*02*,*2*.*07)*	*2*.*24(1*.*58*,*3*.*17)*	*2*.*47(1*.*64*,*3*.*73)*	*2*.*18(1*.*57*,*3*.*03)*
Unknown	*1*.*98(1*.*46*,*2*.*69)*	1.19(0.83,1.71)	*2*.*08(1*.*48*,*2*.*94)*	*2*.*08(1*.*38*,*3*.*16)*	*2*.*46(1*.*74*,*3*.*48)*
**Stage**					
Localized	Reference	Reference	Reference	Reference	Reference
Regionalized	*3*.*43(2*.*62*,*4*.*49)*	*2*.*55(1*.*96*,*3*.*31)*	*3*.*59(2*.*81*,*4*.*60)*	*4*.*28(3*.*25*,*5*.*62)*	*3*.*58(2*.*71*,*4*.*72)*
Distant	*20*.*95(15*.*99*,*27*.*45)*	*16*.*50(12*.*74*,*21*.*37)*	*22*.*50(17*.*55*,*28*.*83)*	*21*.*96(16*.*80*,*28*.*70)*	*21*.*17(16*.*06*,*27*.*90)*
Unknown	*3*.*59(2*.*36*,*5*.*45)*	*5*.*25(3*.*50*,*7*.*88)*	*6*.*79(4*.*97*,*9*.*27)*	*5*.*18(3*.*60*,*7*.*45)*	*5*.*64(3*.*73*,*8*.*51)*
**Primary site** ^c^					
Left	Reference	Reference	Reference	Reference	Reference
Right	0.86(0.73,1.02)	0.90(0.76,1.06)	1.01(0.87,1.17)	1.15(0.97,1.36)	0.93(0.79,1.09)
**Treatment modality**					
**Chemotherapy**					
No/Unknown	Reference	Reference	Reference	Reference	Reference
Yes	1.02(0.85,1.22)	*0*.*80(0*.*66*,*0*.*97)*	*0*.*78(0*.*66*,*0*.*93)*	*0*.*71(0*.*59*,*0*.*86)*	0.91(0.76,1.09)
**Radiation**					
No/Unknown	Reference	Reference	Reference	Reference	Reference
Yes	0.96(0.76,1.22)	1.03(0.81,1.32)	1.07(0.84,1.37)	1.04(0.80,1.35)	1.10(0.85,1.42)

Abbreviation: CRC, colorectal cancer; HR, hazard ratio; NA, non-applicable.

Notes: 1) Italicized text indicates statistically significant result; 2) All model was adjusted for year of diagnosis (variable not shown).

^a^ Others include divorced, separated, and widow.

^b^ Grade 1: Well differentiated; Grade 2: moderately differentiated; Grade 3: poorly differentiated; Grade 4: undifferentiated.

^c^ Right: cecum to transverse; left: splenic flexure to rectum.

^d^ Rurality was not included due to Clayton is considered as urban.

## Discussion

Findings from our study elucidate that geographic regions have significantly different CRC survival rates in the state of Georgia. We observed that the five-year CRC survival rates were lowest in Clayton County (65.9%) compared to the West Central, East Central, Southeast, and Northeast regions (p-value <0.05). Clayton residents possibly experience barriers to the access of appropriate healthcare resources because 18% of Clayton residents reported having no health insurance, compared with 14% of Georgians as a whole [20, 21]. Remarkably, this region has lower socio-economic resources in Georgia, with 19% of Clayton residents reporting living below the poverty level compared to 14% of Georgians, overall [20]. Because there are no studies that examined the relationship of place-based difference in CRC survival rates within these five regions of Georgia, it is impossible to directly discuss our results with prior literature. Yet, a Nevada study with similar demographic profiles to Georgia (e.g., lack of health care insurance or low socioeconomic communities) demonstrated the place-based differences in CRC survival, with significant lower CRC survival observed in Southern Nevada compared to Northwestern Nevada [22]. Further, another study focusing on regional differences between different countries outside of the US in CRC survival reported that although the 5-year survival rate has increased to over 60% in the last decade, the CRC survival rates are significantly worse in Australia, Canada, Denmark, Norway, and Sweden compared to the United Kingdom (UK) [15, 23, 24]. Place-based differences in CRC survival rates may be due to unique geographic and sociodemographic characteristics, such as high proportion of ethnic minorities, poverty, and uninsured rates as well as public awareness of cancer. These disparities may greatly impact CRC survival outcome due to unequal access to CRC screening facilities and optimal diagnostics and specialist care, which may lead to later-stage of CRC diagnosis [25].

In our multivariable analysis, we also found that the Georgians living in Clayton County had an 11% increased risk of death compared to Georgians living in the West Central region when adjusting for demographics, tumor characteristics, and treatment modality. As discussed before, this disparity may be because Clayton region considers as a lower socio-economic area with significant higher poverty level compared to Georgia as a whole (19% vs. 14%). Further, 18% of Clayton residents reported having no health insurance which may greatly impact the accessibility of cancer treatment and screening resources [20, 21]. Our findings are partially consistent with a prior study in Georgia using the 1999–2019 mortality data from Centers for Disease Control and Prevention (CDC), wherein Moore et al (2022) reported that hot spots counties for CRC mortality in Georgia include the north-eastern Piedmont region to eastern Coastal Plains region; and another cluster in southwestern Georgia [17]. However, Moore and colleagues did not find Clayton County as a hot spot county in CRC death. This difference may be due to their use of an ecological study design using county-level data, which limited their ability to draw conclusions about CRC deaths at the individual level. A mentioned Nevada study also demonstrated the place-based differences in CRC survival. Callahan and colleague reported that Southern Nevadans were at 17% higher risk of death than residents living in Northwestern Nevada [22]. Because place-based differences in CRC survival have been reported by a few studies, this highlights the need for investigating multifaceted factors on why the location differences were associated with CRC survival, particularly for the high burden of CRC mortality regions.

When exploring demographic characteristics, we found that age at diagnosis, gender, race, and marital status were associated with CRC survival (p-value <0.05). Age at diagnosis continues to be a key modifiable factor related to CRC survival in our analysis regardless of regions. Consistent with a prior study, CRC patients with older age at diagnosis are known to have shorter overall survival [26]. We found that CRC patients aged 75+ years had worse survival than those in the younger groups (18–44 years). It is possible that older patients were more likely to discontinue treatment for CRC due to side effects or the burden of comorbid diseases [27]. Consistent with a prior study, Gullickson et al reported that patients diagnosed with CRC between the ages of 50 and 69 years had greater survival than those in the older groups [28]. Another important finding in our result is that CRC patients living in the Clayton County seem to have more early-onset CRC diagnosis with 8.8% of those diagnosed at age 18–44 compared to other regions of Georgia. Further, we found that older Clayton Georgians (≥ 45 years) were associated with reduced CRC death compared to 18–44 age groups despite not being statistical significance in our adjusted analyses. These findings may suggest a higher burden of undetected preclinical early-onset CRC among younger Clayton Georgians. Targeted awareness campaigns regarding CRC risk should be prioritized for adults <45 years in Clayton regions. Our this finding also have implications on whether to decrease the age of initial CRC screening given that the current U.S. Preventive Services Task Force (USPSTF) recommended starting CRC screening at age 45 years [29].

In addition, we found that women had 15%-22% lower risk of CRC death in the Clayton, East Central, Southeast, and Northeast regions. Several explanations for the survival advantage of women for most cancers have been reported, including gender-differences in risk factor prevalence, comorbidities, and/or health seeking behaviors [30, 31]. Married patients in Georgia, particularly in the West Central, East Central, Southeast, and Northeast regions, had greater CRC survival than single patients. This phenomenon is often linked to greater social support and financial benefits [32, 33]. Lastly, race differences in CRC survival were also observed in our analysis. We found that Black CRC patients had worse CRC survival comparing to White patients, particularly among Clayton, West Central, and Northeast Georgians. These disparities may be due to differences in access to care, cancer screening, and other sociodemographic factors as well as etiology [34].

For tumor characteristics, stage at diagnosis and tumor grade are well-established predictors of CRC mortality [35–37]. Haggar et al reported that patients with distant stage CRC were at 20-fold increased risk of CRC related death [36]. Having high-grade tumors were associated with increased risk of CRC death [37]. Our multivariable analyses also confirmed these associations regardless of regions of Georgia. Late stage at diagnosis and high-grade tumors related to worse CRC survival is likely attributed to multiple factors, including absence of programs for early diagnosis or screening [38], limited access to adequate care [39], and scarcity of appropriately trained professionals, which may affect care utilization in some communities [40].

Finally, we found that CRC patients who received chemotherapy had lower risk of CRC death in the West Central, East Central, and Southeast regions, which is consistent with prior studies [9, 41]. It is possible that those CRC patients were treated with more aggressively due to more advanced tumors. Evidence shown that using adjuvant therapy is one of modifiable factors related to greater CRC survival [26, 41, 42]. For example, the use of adjuvant chemotherapy after curative surgery for CRC patients were associated with higher CRC survival with significantly improved the prognoses compared to the non-chemotherapy group [41, 42]. However, we were unable to control sequence of systemic therapy due to unavailable information from the 1975–2016 SEER program.

A major strength of this study is the multi-year data with large sample size provided by the SEER program, enabling the creation of stable survival analysis by the five regions of Georgia for comparison purposes. Our findings are critical to guide local public health initiatives for CRC early detection. One of modifiable factors with the potential for leading to better CRC outcomes is CRC screening because it can detect tumors at earlier stages as well as removal of pre-cancerous polyps [43]. More importantly, additional resources should be committed to the development of CRC education programs to improve awareness among Clayton residents. Despite its strengths, there are a few limitations that should be noted. First, individual-level data was not available for other sociodemographic characteristics and lifestyle factors as cancer registries usually do not collect this information. Factors, such as low income, physical inactivity, current smoker, and heavy alcohol use, may greatly increase the risk of CRC mortality [44–46]. Second, the presence of comorbidities is also not available from the SEER program. Research has shown that patients with multiple comorbidities have worse survival outcomes [43]. Third, we are unable to examine tumor markers and/or molecular subtypes that negatively impact CRC survival. More importantly, individual-level CRC screening history is also not available from cancer registries. Timely CRC screening uptake may make a significant impact on reducing CRC mortality [47]. Finally, our study was limited to the selected five regions in Georgia, which may limit availability to generalize results to different populations in other geographic areas. Using cancer registries may also have potential biases due to underreporting of outcomes when patients leave the registry or are not adequately followed up. Therefore, findings from our study suggest that future research integrated multifactorial factors (e.g., comorbidities, family/personal history of cancer, lifestyle factors, CRC screening use) through patient medical records or self-administered health survey may further elucidate the relationship between our identified predictors with CRC survival. More studies are also needed to examine the impact of area-level barriers (e.g., availability of screening and treatment resources) on CRC outcomes in Georgia.

## Conclusions

We observed place-based differences in CRC survival in Georgia. The lowest 5-year survival rate was found among Georgians living in Clayton County compared to Georgians living in the West Central, East Central, Southeast, and Northeast regions. Among Georgians living in Clayton County, risk factors associated with higher risk of CRC death included older age at diagnosis, high-grade tumors, and distant stage. Future research is needed to determine the place-based barriers for CRC survival. All relevant stakeholders, including clinicians and researchers, must strategically and aggressively approach any opportunities for improving primary prevention of CRC through timely CRC screening and maximizing the survival potential for the most impacted regions of Georgia. Approaches, such as culturally tailored CRC screening interventions may improve CRC early detection and outcomes.
